# Supplementary material for: Genomic Variability of SARS-CoV-2 Omicron Variant Circulating in the Russian Federation during Early December 2021 and Late January 2022
Source: Pathogens. 2022 Dec 2;11(12):1461. doi: 10.3390/pathogens11121461 (PMC9786029; doi:10.3390/pathogens11121461)

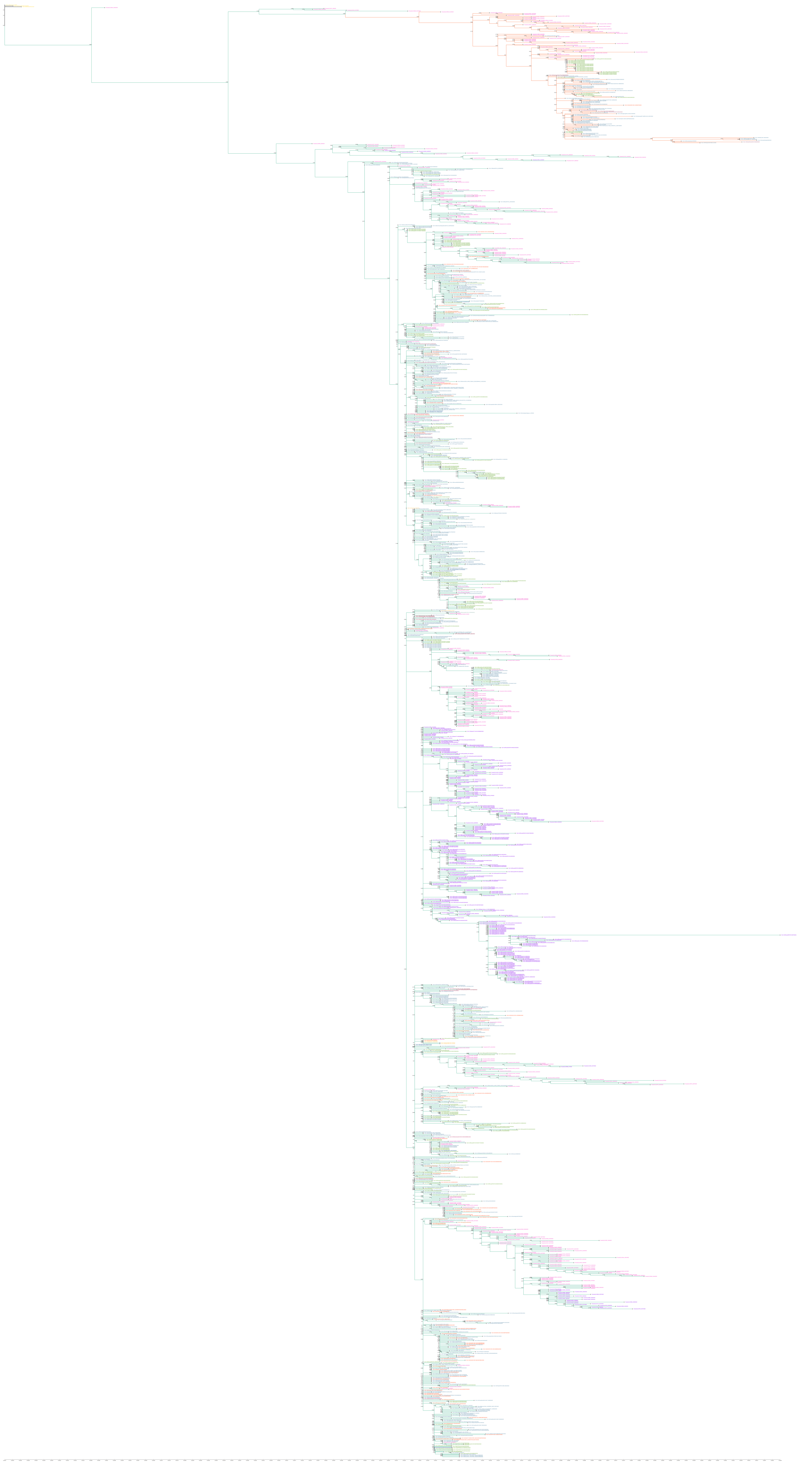

Supplement: Supplementary file 1 [file Figure S1. Phylogenetic tree built using Maximum-Likelihood method including Russian FMBA and GISAID datasets and global subsampled GISAID dataset. BA.1 cluster .pdf › Figure S1. Phylogenetic tree built using Maximum-Likelihood method including Russian FMBA and GISAID datasets and global subsampled GISAID dataset. BA.1 cluster .pdf]
