# Supplementary material for: Genomic Variability of SARS-CoV-2 Omicron Variant Circulating in the Russian Federation during Early December 2021 and Late January 2022
Source: Pathogens. 2022 Dec 2;11(12):1461. doi: 10.3390/pathogens11121461 (PMC9786029; doi:10.3390/pathogens11121461)

Supplementary figures.

Figure S1. Phylogenetic tree built using Maximum-Likelihood method including Moscow and Russian GISAID datasets and global subsampled GISAID dataset. BA.1 cluster branches are marked with green color, BA.2 branches – with orange color.

Identificators of originating countries:

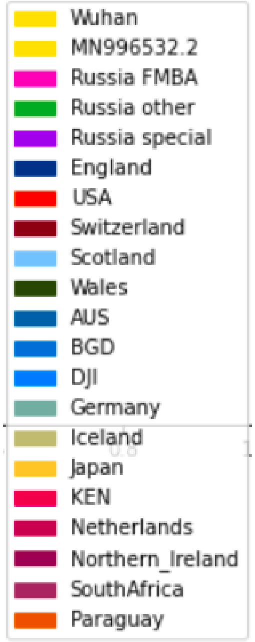

Figure S2. Phylogenetic analysis of the Russian SARS-CoV-2 Omicron strains and closely related viral variants extracted from UShER database to identify clusters with global Omicron viral variants related with the Russian isolates. The tree has Orange branches related with BA.2 variant and light green branches related with BA.1 variants.

Identificators of originating countries:

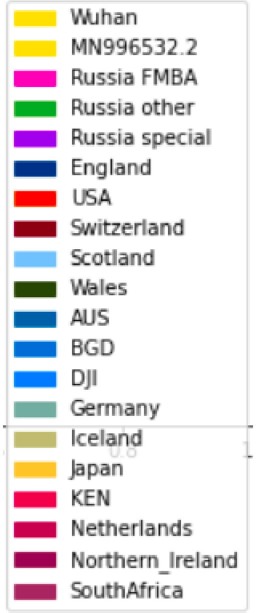

Supplement: Supplementary file 1 [file pathogens-11-01461-s001.zip › Sipplementary figures.pdf]
